# Adherence to endodontic treatment protocols in the clinical practice of Lithuanian dentists and endodontists—cross-sectional study

**DOI:** 10.1186/s12903-026-09105-9

**Published:** 2026-07-02

**Authors:** Eigile Barsyte, Tadas Venskutonis

**Affiliations:** Department of Dental and Oral Pathology, Lithuanian University of Health Sciences, Eivenių g. 2, Kaunas, LT-50028 Lithuania

**Keywords:** Endodontics, Root Canal Therapy, Practice Guidelines as Topic, Root Canal Irrigants, Root Canal Obturation

## Abstract

**Objective of the study:**

To evaluate contemporary endodontic practice patterns among general dental practitioners and endodontists in Lithuania, compare the clinical protocols used, and assess their adherence to international endodontic guidelines.

**Materials and methods:**

A cross-sectional survey was conducted among Lithuanian general dental practitioners and endodontists. The questionnaire addressed major stages of endodontic treatment, from isolation and canal preparation to obturation and post-treatment restoration. A total of 470 participants completed the survey (372 general dental practitioners, 77 endodontists, and 20 “other” (did not specify their professional category) responses), of whom 450 were included in the final analysis. Data were analyzed using descriptive and inferential statistics. Descriptive statistics were calculated for categorical variables and presented as frequencies and percentages. Associations between groups were assessed using the chi-square (χ^2^) test, logistic regression and Spearman correlation analysis. Degrees of freedom (df) and corresponding *p*-values were reported, with statistical significance set at *p* < 0.05.

**Results:**

Rubber dam was used in all endodontic procedures by 101 (34.4%) of general practitioners and 65 (84.4%) of endodontists. An electronic apex locator was used by the vast majority of both general practitioners and endodontists for working length determination. Sodium hypochlorite was the most commonly used irrigant; general practitioners favored passive irrigation activation, whereas endodontists more frequently employed ultrasonic and sonic activation methods. Calcium hydroxide was the most frequently used intracanal medicament. Cold lateral condensation was more commonly used by general practitioners, while endodontists preferred warm vertical compaction and single-cone obturation techniques. Private practice was associated with higher odds of bioceramic cement use. Direct composite restorations were the most common post-endodontic restorations, with crowns used less frequently.

**Conclusion:**

Compared to data from 2010, endodontic practice in Lithuania has shown greater adherence to European Society of Endodontology (ESE) and the American Association of Endodontists (AAE) guidelines. Endodontists consistently apply evidence-based methods more rigorously than general practitioners. However, insufficient rubber dam usage and variable implementation of the latest clinical protocols indicate that guideline adherence is still not routine. These findings underscore the importance of continuous professional development and application of updated recommendations in clinical practice to ensure high-quality endodontic care and patient safety.

## Introduction

Endodontic treatment is one of the most complex and precision-demanding procedures in dentistry [1]. This field encompasses the diagnosis and management of pulpitis, periapical pathologies, and other diseases of the pulp and root canal system. In 1984, Vertucci conducted one of the first systematic studies on the anatomy of permanent human teeth, documenting significant variability in canal morphology, including additional canals, curvatures, and unusual root configurations. He emphasized that such complex anatomy can complicate root canal instrumentation and disinfection, directly affecting treatment success [2].

The outcome of treatment also depends on the clinician’s competence and ability to strictly follow established protocols, which influences tooth survival and long-term procedural success [3]. The European Society of Endodontology (ESE) provides clear, evidence-based diagnostic and treatment algorithms, including patient history taking, clinical and radiographic examination, maintenance of asepsis, canal instrumentation, irrigation, disinfection, obturation, and follow-up recommendations [4]. Adherence to these protocols is directly associated with improved treatment prognosis, lower complication rates, and higher long-term tooth survival [5].

Although the first documented attempts at root canal treatment date back to 1728, when Pierre Fauchard described pulp removal and filling with lead [6], modern endodontics relies on detailed international protocols covering asepsis, instrumentation, disinfection, and obturation [7]. Despite centuries of practice, not all clinicians consistently follow contemporary guidelines in everyday clinical settings. Literature indicates that general dental practitioners do not always adhere to recommended endodontic standards [8]. The most common deviations include insufficient use of irrigants [9], inadequate canal preparation depth [10], failure to use a rubber dam [11], inappropriate instrument selection [12], and non-compliance with follow-up radiographic evaluation protocols [13]. Such deviations from evidence-based guidelines may lead to increased retreatment needs, treatment failures, and poorer long-term outcomes [14].

Endodontists with specialized training generally adhere more rigorously to recommended clinical protocols due to more intensive education, deeper theoretical and practical knowledge, and greater daily clinical experience in root canal therapy [15–17]. In contrast, general dental practitioners perform a wider range of procedures, making their endodontic practices more heterogeneous [18]. In Lithuania, there is still a lack of research evaluating how general dentists and endodontists follow contemporary endodontic treatment protocols, although such data are essential for identifying practice gaps, planning professional training, and improving patient care. A study conducted in Lithuania in 2010 assessing the technical aspects of root canal treatment among dentists found that 66% of respondents had never used a rubber dam in daily practice [19]. These findings highlight the need to strengthen professional development and continuing education among local specialists.

A national evaluation should therefore identify which treatment stages pose the greatest challenges, which protocol steps are skipped or inadequately performed, and whether significant differences exist across clinician qualifications, age groups, or practice settings.

Based on differences in education and clinical specialization, we hypothesized that there would be variations in adherence to endodontic protocols between the two groups.The aim of this study was to assess how Lithuanian general dental practitioners and endodontists adhere to internationally recommended endodontic treatment protocols in clinical practice and to identify factors associated with the application of recommended practices.

## Material and methods

### Study design

A cross-sectional study was conducted at the Lithuanian University of Health Sciences (LSMU), Kaunas, Lithuania, from October 10, 2025, to November 24, 2025.

### Materials and subjects

#### Ethics approval

This survey was reviewed by the Lithuanian Bioethics Committee, which issued a document confirming that no bioethics approval was required for this study (No. 6B-25–19).

#### Consent to participate

Informed consent was obtained from all respondents through voluntary completion of the survey.

The study included individuals holding valid general dental practitioner or endodontist licenses and actively practicing in their profession. Eligibility was determined based on self-reported data provided by respondents, as no external verification of professional status was performed due to the survey-based study design. The survey was posted on the website of the Lithuanian Chamber of Dentists. Additionally, the Lithuanian Endodontic Society shared the survey in their newsletter. The questionnaire consisted of short, multiple-choice, and open-ended questions. The questionnaire was developed based on previously published literature [19] and current European Society of Endodontology (ESE) guidelines. No pilot testing was performed. The survey was conducted anonymously, and no identifying personal data were collected. As the categorization of open-ended responses was performed by a single researcher, a degree of subjectivity cannot be excluded, which may have introduced classification bias. The findings are based on self-reported data, which may be influenced by reporting bias and may not accurately reflect actual clinical practice. The distribution of the survey through online channels and the unknown number of individuals who received or viewed it may have introduced selection bias.

### Variables

The main outcomes were adherence to endodontic treatment protocols, including rubber dam use, working length determination, instrumentation, irrigation and disinfection, root canal obturation and post-obturation procedures, and restorative assessment and final restoration. The main exposure variables were professional qualification (general dental practitioner or endodontist), workplace type (private or public), age, and years of clinical experience. Potential predictors may include years of clinical experience and workplace type.

Clinical trial number: not applicable.

### Determination of the study sample

The sample size (the number of specialists required to complete the questionnaire) was calculated using Paniotto’s formula with 95% confidence intervals:

$$\mathrm{n}=1/\left(\triangle^{2}+1/\mathrm{N}\right)$$

Where n is the sample size (number of specialists to be surveyed), Δ is the sampling error (a standard error of 5%, corresponding to a 0.95 probability), and N is the total number of professionals in the study population.

According to the data provided by the State Health Care Accreditation Agency under the Ministry of Health of the Republic of Lithuania (https://vaspvt.lrv.lt/), there are currently 3884 actively licensed general dental practitioners and 96 licensed endodontists in Lithuania. Therefore, the required sample size was determined to be 363 general dental practitioners and 77 endodontists. The calculated sample size was achieved.

The total number of doctors who received the questionnaire is unknown due to open online distribution, therefore, the response rate cannot be calculated.

### Statistical analysis

The responses obtained from participants were entered into Microsoft Excel® 2019 (Microsoft Corp., Seattle, WA, USA) and analyzed using IBM SPSS® Statistics version 30.0 (IBM Corp., Armonk, NY, USA).

All survey items were mandatory, therefore, complete responses were obtained from all participants, and no missing data handling procedures were required.

Open-ended responses were reviewed and coded into thematic categories based on content similarity. Responses that did not fit into predefined categories were assigned to an “other” category and excluded from the present analysis.

Descriptive statistics were calculated for categorical variables and presented as frequencies and percentages. The chi-square (χ^2^) test, logistic regression and Spearman correlation were used to assess associations between categorical variables. Degrees of freedom (df) and corresponding *p*-values were reported.

A *p*-value of < 0.05 was considered statistically significant.

Sensitivity analyses were not conducted.

The study is reported in accordance with the STROBE (Strengthening the Reporting of Observational Studies in Epidemiology) guidelines.

## Results

### Study participants

A total of 470 individuals participated in the survey, including 77 (16.4%) endodontists, 372 (79.1%) general dental practitioners, and 20 (4.3%) respondents who selected “other”. The “other” category included respondents who did not identify themselves as general dental practitioners or endodontists. Respondents who selected the “other” category were excluded from the statistical analysis. Of the 372 general dental practitioners, 294 (79.2%) perform endodontic treatment. The median age of the respondents was 39 years (SD 11.093; range: 23–76). Age distribution was as follows: 65 (13.9%) were younger than 30 years, 145 (30.9%) were 31–40 years old, and 161 (34.3%) were older than 40 years (Table 1). Most participants had 11–20 years of professional experience (Table 2). Analysis of workplace types revealed a statistically significant difference in the number of endodontists working in private clinics (p = 0.001) (Table 3).

**Table 1:** Age distribution among respondents of different genders

Age group (years)	Male	Female
≤ 30	8 (13.8%)	57 (18.2%)
31–40	15 (25.9%)	130 (41.5%)
> 40	35 (60.3%)	126 (40.3%)

χ^2^- 8.171, df – 2, *P*-value = 0.017

**Table 2:** Distribution of endodontists and general practitioners according to work experience

Years of work experience	Specialization	
	Endodontists *N* (%)	General dentists *N* (%)
< 5	9 (11.7%)	41 (13.9%)
6–10	17 (22.1%)	68 (23.1%)
11–20	34 (44.2%)	77 (26.2%)
21–30	15 (19.5%)	66 (22.4%)
> 30	2 (2.6%)	42 (14.3%)

χ^2^- 14.118, df – 4, *P*-value = 0.007

**Table 3:** Distribution of endodontists and general dentists in different types of workplaces

Type of workplace	Specialization	
	Endodontists *N* (%)	General dentists *N* (%)
Private clinic	65 (84.4%)	184 (62.6%)
Public clinic	4 (5.2%)	44 (15.0%)
Both privately and at a state clinic	8 (10.4%)	66 (22.4%)

χ^2^- 13.285, df – 2, *P*-value = 0.001

### Use of rubber dam

Statistically significant differences were observed regarding the consistent use of rubber dam between dentists working in private and public institutions (p < 0.001). Most respondents working in public institutions reported that they never use a rubber dam during endodontic treatment 21 (43.8%) (Table 4). The majority of endodontists 65 (84.4%) always use a rubber dam, whereas general dental practitioners most frequently reported either “always” 101 (34.4%) or “never” 80 (27.2%) (Table 5). Consistent use of a rubber dam was most frequently reported by younger respondents and those with less professional experience (58.5%). Respondents over 40 years of age most often reported never using a rubber dam (32.9%). Respondents who indicated using a rubber dam “often,” “sometimes,” “rarely,” or “never” were asked to specify the reasons for not using it consistently. (Table 6).

**Table 4:** Use of rubber dam system in different types of workplaces

Use of rubber dam	Type of workplace		
	Private clinic *N*(%)	Public clinic *N* (%)	Both privately and at a state clinic *N* (%)
Always	138 (55.4%)	7 (14.6%)	21 (28.4%)
Often	33 (13.3%)	2 (4.2%)	11 (14.9%)
Sometimes	29 (11.6%)	7 (14.6%)	14 (18.9%)
Rarely	13 (5.2%)	11 (22.8%)	5 (6.8%)
Never	36 (14.5%)	21 (43.8%)	23 (31.1%)

χ^2^- 62.105, df – 8, *P*-value < 0.001

**Table 5:** Use of rubber dam between endodontists and general practitioners

Use of rubber dam	Specialization	
	Endodontists *N* (%)	General dentists *N* (%)
Always	65 (84.4%)	101 (34.4%)
Often	8 (10.4%)	38 (12.9%)
Sometimes	4 (5.2%)	46 (15.6%)
Rarely	0 (0%)	29 (9.9%)
Never	0 (0%)	80 (27.2%)

χ^2^- 67.998, df – 4, *P*-value < 0.001

**Table 6:** Reasons for not using a rubber dam among general dentists and endodontists

Reasons for avoiding rubber dam isolation	Endodontists *N* (%)	General dentists *N* (%)
No need	2 (16.7%)	24 (21.6%)
Lack of time	1 (8.3%)	40 (36.0%)
Interference	4 (33.3%)	7 (6.3%)
Does not provide workspace	1 (8.3%)	9 (8.1%)
Lack of skill to place	0 (0%)	14 (12.6%)
Anterior teeth	3 (25.0%)	10 (9.0%)
Patient’s preference	1 (8.3%)	7 (6.3%)

χ^2^- 15.667, df – 6, *P*-value = 0.016

### Working length determination

Most Lithuanian dentists always use an apex locator to determine working length: 76 (98.7%) of endodontists and 275 (93.5%) of general dental practitioners. Only 1 (1.3%) endodontist and 19 (6.5%) general dental practitioners did not consistently use this method. Working length was checked multiple times during the procedure by 72 (93.5%) of endodontists and 240 (81.6%) of general dental practitioners. 58 (75.3%) of endodontists and 224 (60.2%) of general dental practitioners use radiography as an adjunct method, while 33 (42.9%) of endodontists and 153 (41.1%) of general dental practitioners use radiographs to adjust working length based on file position (Table 7).

**Table 7:** Additional working length determination methods used by endodontists and general dentists

Working length determination methods	Endodontists *N* (%)	General dentists *N* (%)	χ^2^	df	*P*-value
Radiograph	58 (75.3%)	224 (60.2%)	37.579	2	< 0.001*
Endodontic file on radiograph	33 (42.9%)	153 (41.1%)	18.810	2	0.001*
Tactile method	14 (18.2%)	64 (17.2%)	4.211	2	0.122
Paper point method	5 (6.5%)	55 (14.8%)	6.996	2	0.030*
Radiograph with gutta-percha in the canal	0 (0%)	3 (0.8%)	0.789	2	0.674
No additional methods used	3 (3.9%)	2 (0.5%)	7.047	2	0.029*

^*^Statistically significant (*P* < 0.05), degree of freedom for the chi-square (χ2)*. N* = number

### Instrumentation, irrigation, and disinfection

For root canal irrigation, specialists most frequently used sodium hypochlorite, EDTA, and distilled water (Table 8). No statistically significant association was found between workplace type and the choice of irrigating solution. A negative Spearman correlation was observed between respondents with 11–20 years of experience and citric acid usage (ρ =—0.071). The most commonly used irrigation sequences were: NaOCl throughout the entire treatment, followed by a chelating agent (EDTA or citric acid) for 1–2 min at the end, or NaOCl → EDTA → NaOCl protocol (Table 9). Endodontists more frequently used rotary instruments and ultrasonic activation, whereas general dental practitioners tended to use manual instruments. A positive correlation was observed between lower work experience and the use of rotary instruments (ρ = 0.079).

**Table 8:** Solutions selected by clinicians

Irrigation solutions	Endodontists *N* (%)	General dentists *N* (%)	χ^2^	df	*P*-value
NaOCl	75 (97.4%)	285 (76.6%)	84.453	2	< 0.001*
Chlorhexidine	29 (37.7%)	113 (30.4%)	10.676	2	0.005*
Hydrogen peroxide	0 (0%)	9 (2.4%)	2.393	2	0.302
Water	60 (77.9%)	174 (46.8%)	45.559	2	< 0.001*
EDTA	76 (98.7%)	224 (60.2%)	78.080	2	< 0.001*
NaOCl + HEDP (Dual rinse)	6 (7.8%)	11 (3%)	5.055	2	0.08
A mixture of doxycycline and citric acid (Biopure MTAD)	2 (2.6%)	5 (1.3%)	0.998	2	0.607
Citric acid	19 (24.7%)	100 (26.9%)	7.267	2	0.026*
0.9% NaCl	1 (1.3%)	5 (1.3%)	0.272	2	0.873

^*^Statistically significant (*P* < 0.05), degree of freedom for the chi-square (χ2). *N* = number

**Table 9:** Choices of irrigation solution protocols among general dentists and endodontists

Irrigation solution protocols	Endodontists *N* (%)	General dentists *N* (%)	χ^2^	df	*P*-value
Only sodium hypochlorite (NaOCl) throughout the entire treatment	2 (2.6%)	60 (16.1%)	13.365	2	0.001*
NaOCl throughout the entire treatment, followed by a chelating agent (EDTA/citric acid) at the end (1–2 min)	42 (54.5%)	162 (43.5%)	19.221	2	< 0.001*
NaOCl → chelating agent (EDTA/citric acid) → NaOCl	34 (44.2%)	61 (16.4%)	35.737	2	< 0.001*
Use of a chelating agent (EDTA/citric acid) also between instrumentation phases	4 (5.2%)	14 (3.8%)	1.188	2	0.552
Distilled water is used between the irrigants	44 (57.1%)	153 (41.1%)	21.846	2	<.0001*
NaCl solution is used between the irrigants	0 (0%)	9 (2.4%)	2.393	2	0.302
Other	2 (2.6%)	6 (1.6%)	0.731	2	0.694

^*^Statistically significant (*P* < 0.05), degree of freedom for the chi-square (χ2). *N* = number

During instrumentation, 157 (53.4%) of general dental practitioners reported occasionally using a lubricant, whereas 45 (58.4%) of endodontists reported never using one. In both groups, the most commonly used lubricant was RC-Prep (Table 10).

**Table 10:** Choices of chelating lubricants among endodontists and general dentists

	Endodontists *N* (%)	General dentists *N* (%)	χ^2^	df	*P*-value
RC-Prep	31 (40.3%)	210 (56.5%)	28.777	2	< 0.001*
Glyde	1 (1.3%)	6 (1.6%)	0.359	2	0.836
File-Eze	3 (3.9%)	11 (3%)	0.837	2	0.658
EndoGel	1 (1.3%)	7 (1.9%)	0.492	2	0.782
Provided by the workplace	0 (0%)	3 (0.8%)	0.787	2	0.675

^*^Statistically significant (*P* < 0.05), degree of freedom for the chi-square (χ2). *N* = number

Calcium hydroxide as an intracanal medicament between visits was used by 282 (96.2%) of general dental practitioners and 76 (98.7%) of endodontists. Overall, 303 (84.6%) of specialists used pre-prepared paste in a syringe, while 55 (15.4%) mixed calcium hydroxide before placement into the canal. Endodontists most frequently used calcium hydroxide in cases of acute periapical inflammation 65 (84.4%) or extensive abscesses/fistulas 62 (80.5%). General dental practitioners typically applied calcium hydroxide between visits for additional disinfection 226 (60.8%) or after instrumentation as a temporary medicament 197 (53%).

### Root canal obturation and post-obturation procedures

For long-term root canal obturation, general dental practitioners more frequently chose cold lateral condensation, whereas endodontists preferred warm vertical or single-cone techniques. Less than half of general dental practitioners 168 (45.2%) disinfected gutta-percha, while the majority of endodontists 70 (90.9%) performed this step consistently. The most commonly used disinfectants were NaOCl 136 (57.1%) and alcohol 77 (32.4%). Most endodontists 74 (96.1%) used hydraulic calcium silicate cement, whereas general dental practitioners used resin-based sealers 163 (43.8%). Logistic regression analysis indicated that practice in private clinics is associated with higher odds of using bioceramic cements (OR 8.352 [4.004–17.423]) (Table 11). Disinfection typically involved sodium hypochlorite or alcohol, with no significant differences between groups. The most commonly used post-obturation temporary material was glass ionomer, chosen by 157 (42.2%) of general dental practitioners and 26 (36.4%) of endodontists.

**Table 11:** Univariable logistic regression analysis of factors associated with workplace type and protocol characteristics (only statistically significant results shown)

Variable	Public clinic *N* (%)	Private clinic *N* (%)	OR [95% CI]
Citric acid	19 (18.8%)	110 (34.1%)	2.238 [1.025–4.787]
Distilled water	19 (39.6%)	178 (55.1%)	1.874 [1.009–3.478]
Rotary instruments	18 (66.7%)	181 (88.3%)	3.771 [1.523–9.334]
Calcium hydroxide in acute periapical inflammation	26 (54.2%)	224 (69.3%)	1.915 [1.035–3.541]
Calcium hydroxide in the presence of extensive abscess (phlegmon) or a fistula	20 (41.7%)	206 (63.8%)	2.465 [2.330–4.569]
Calcium hydroxide in cases of a wide apex	7 (14.6%)	96 (29.7%)	2.477 [1.073–5.717]
Calcium hydroxide in the presence of root apex resorption	8 (16.7%)	110 (34.1%)	2.582 [1.168–5.708]
Use of bioceramic sealer	10 (20.8%)	222 (68.7%)	8.352 [4.004–17.423]
Use of zinc oxide eugenol–based sealer	16 (33.3%)	40 (12.4%)	0.283 [0.142–0.561]
Delayed tooth restoration after several weeks	22 (45.8%)	100 (31.0%)	0.530 [0.287–0.980]
Passive use of irrigating solutions	33 (68.8%)	150 (46.4%)	0.394 [0.206–0.754]
Ultrasonic activation of irrigating solutions	6 (12.5%)	121 (37.5%)	4.193 [1.731–10.155]

Vital pulp therapy was generally performed only in selected cases, such as in young patients or when accidental pulp exposure occurred. This approach was reported by 152 (51.7%) of general dental practitioners and 35 (45.5%) of endodontists. Half of general dental practitioners never perform revitalisation procedures in cases of wide open pulp chambers, whereas 69 (89.6%) of endodontists do. Additionally, 57 (74%) of endodontists never use devitalising paste, compared with fewer than half 142 (48.3%) of general dental practitioners.

### Restorative assessment and final restoration

Before treatment, 361 (97.3%) of doctors assessed the prognosis of restoration, with no statistically significant difference between endodontists and general dental practitioners. Dentists also significantly more frequently removed old restorations and carious lesions prior to initiating treatment. For final restoration after obturation, 73 (96.1%) of endodontists referred the patient to a prosthodontist or general dentist, whereas 172 (46.2%) of general dental practitioners performed the final restoration themselves within a few days. Dentists of both specializations preferred to restore with composite first, followed by crowns.

## Discussion

The results of this study provide insights into endodontic treatment practices among general dental practitioners and endodontists in Lithuania. The findings allow for a comparison of treatment protocols and methodologies both between different specialties and with data from other countries, as well as international guidelines.

101 (34.4%) of general dental practitioners and 65 (84.4%) of endodontists reported using a rubber dam in all cases of endodontic treatment, indicating greater professionalism among endodontists and stronger awareness of its importance for treatment success. For example, in a study conducted in Lithuania in 2010, only 11.8% of dentists used a rubber dam consistently [19]. In comparison, in Saudi Arabia 21.6% of general dental practitioners used a rubber dam [20], in Turkey 16.7% of dentists chose to use a rubber dam for root canal treatment [21], and in China, according to Zou et al., approximately 63% of respondents used a rubber dam, although consistent use in restorative or endodontic procedures was low [22]. These results indicate that more general dental practitioners in our country adhere to international guidelines than in other countries. Nevertheless, the use of the rubber dam among both dentists and endodontists remains insufficient. According to the American Association of Endodontists (AAE, 2024), rubber dam use is considered the “standard” and an essential component of any nonsurgical endodontic treatment. Its use is mandatory to prevent microbial contamination and ensure patient safety [23].

Rubber dam usage was more frequent among practitioners in private clinics than among those in public institutions, suggesting that resource constraints in the public sector may be a contributing factor. Statistically significant differences were found according to professional experience: dentists with less than 5, 6–10, or 11–20 years of experience used a rubber dam significantly more often than those with 21–30 years or more. Thus, rubber dam usage is more common in the clinical practice of newly graduated dentists than in earlier graduates [24]. This may result from curricular changes emphasizing rubber dam use, combined with updated international guidelines that increasingly frame it not as a recommendation, but as a necessity.

Additionally, 14 (11.4%) of respondents indicated lacking the skills to place a rubber dam. Therefore, there are still specialists in our country who need to enhance their qualifications, as theoretical knowledge and practical skills must be continuously updated to comply with contemporary standards [4]. For example, in Saudi Arabia, 40.5% of respondents indicated that the reason for not using a rubber dam was its unavailability in the workplace [20]. These data suggest that in Lithuania, rubber dam non-use is more often associated with subjective factors (time management, work organization, or personal attitudes), whereas in other countries, it is linked to infrastructure or supply issues.

The European Society of Endodontology (ESE) guidelines recommend the consistent use of a rubber dam in all uncomplicated endodontic procedures [4]; therefore, Lithuanian dentists should adhere to these guidelines in daily clinical practice. Other sources indicate that treatment without a rubber dam has significant implications for patient safety and medicolegal responsibility and increases the risk of cross-contamination [25, 26]. For these reasons, there is a need to change practitioners’ attitudes and increase rubber dam usage in our country.

An apex locator was used to determine working length by nearly all endodontists 76 (98.7%) and most general dental practitioners 275 (93.5%) in their daily practice, although no statistically significant difference was found. A study conducted in the United Kingdom showed that 86.4% of endodontists and 53.2% of general dental practitioners used an apex locator [27], which is considerably lower than in Lithuania. Most specialists checked working length multiple times during the procedure. Only 5 (1.1%) did not use any additional method. Literature sources indicate that working length varied between first and second appointments in 34% of patients [28], underscoring that endodontists take a more meticulous approach to canal obturation and procedural error prevention. In the United States, 48% of dentists use radiography, 21% tactile sensation, and 6% paper points [29], suggesting that Lithuanian specialists frequently double-check working length compared to U.S. dentists.

For canal irrigation, endodontists 75 (97.4%) and general dental practitioners 287 (76.6%) most commonly used sodium hypochlorite (NaOCl). The most common irrigation protocols were NaOCl throughout the procedure, followed by a chelating solution (EDTA or citric acid) for 1–2 min at the end, or NaOCl → chelating solution → NaOCl, as recommended by the ESE [7]. Thus, Lithuanian dentists perform canal irrigation according to ESE guidelines. General dental practitioners more often used passive syringe and needle irrigation 178 (47.8%), while endodontists used activation methods: 56 (72.7%) ultrasonic and 36 (46.8%) low-frequency sonic. Although studies show that ultrasonic and sonic irrigation achieve the greatest penetration depth [30], due to methodological heterogeneity, it is impossible to recommend a single method. A meta-analysis demonstrated that regardless of the activation method used, canal content and smear layer removal improved in all cases [31].

Lubricant was always used during instrumentation by 63 (21.4%) of general dental practitioners and 6 (7.8%) of endodontists. As in a study conducted 10 years ago, RC-Prep remains the most commonly used lubricant (Table 10) in Lithuania. The decrease in usage may be due to recent studies showing that RC-Prep does not reduce the risk of dentin defects [32]. Additionally, a 2025 study shows that its clinical benefit is limited, especially if not combined with thorough irrigation. Gel lubricants may increase instrument fatigue and fracture risk [28].

Calcium hydroxide was used inter-appointment as an intracanal medicament by 284 (60.6%) of doctors. Overall, 303 (84.6%) used pre-prepared paste in a syringe. Syringe preparation can ensure uniform material consistency, resulting in a consistent effect throughout the canal. Dentists in our country used calcium hydroxide for indications consistent with most literature sources: acute periapical inflammation [33], additional disinfection between visits [34], wide fistulas/pulp necrosis [35], internal or external root resorption cases [36], and as a temporary material after instrumentation (Table 12). Logistic regression analysis showed that practitioners in private clinics were significantly more likely to use calcium hydroxide in various clinical cases (Table 11).

**Table 12:** Clinical indications for calcium hydroxide use among general dental practitioners and endodontists

	Endodontists *N* (%)	General dentists *N* (%)	χ^2^	df	*P*-value
In the presence of acute periapical inflammation	65 (84.4%)	185 (49.7%)	54.681	2	< 0.001*
In the necrotic stage of pulpitis	31 (40.3%)	149 (40.1%)	13.013	2	0.001*
Between visits, when additional canal disinfection is required	58 (75.3%)	226 (60.8%)	37.742	2	< 0.001*
In the presence of extensive abscess (phlegmon) or a fistula	62 (80.5%)	164 (44.1%)	53.346	2	< 0.001*
In cases of a wide apical foramen	21 (27.3%)	82 (22%)	6.897	2	0.035*
In the presence of root apex resorption (internal or external)	39 (50.6%)	79 (21.2%)	36.333	2	< 0.001*
In the presence of a perforation	18 (23.4%)	35 (9.4%)	15.079	2	< 0.001*
Prophylactically, to prevent bacterial colonization	26 (33.8%)	114 (30.6%)	9.186	2	0.010*
After instrumentation, as a temporary medicament between visits	49 (63.3%)	197 (53%)	25.963	2	< 0.001*
Other: (when there is exudate from the canal)	1 (1.3%)	3 (0.8%)	0.363	2	0.834

^*^Statistically significant (*P* < 0.05), degree of freedom for the chi-square (χ2). *N* = number

Less than half of general dental practitioners 168 (45.2%) disinfected gutta-percha before obturation, whereas 70 (90.9%) of endodontists did so consistently. Although gutta-percha points are aseptic in packaging, they can become contaminated after opening and use. In vitro studies indicate that disinfecting gutta-percha reduces the risk of reinfection during obturation [37]. Disinfection was usually performed with NaOCl or alcohol. Nevertheless, a systematic literature review emphasizes the need to carefully select the type of disinfectant, concentration, and immersion time to preserve gutta-percha quality [38].

General dental practitioners more often used cold lateral condensation 228 (61.3%), whereas endodontists used warm vertical condensation 57 (74%) and the single-cone technique 55 (71.4%). Literature indicates that ideal obturation should be fully compacted, adapt to canal walls, and be tightly sealed to the periodontium [39]. Meta-analyses of in vitro studies report no statistical differences between cold lateral condensation and single-cone techniques; however, warm vertical condensation showed better results compared to cold lateral condensation [40]. Endodontists most frequently used hydraulic calcium silicate cement 74 (96.1%), while general dental practitioners used resin-based sealers 163 (43.8%). Data from 2025 suggest that hydraulic calcium silicate cement, more often used by endodontists, provide better long-term treatment prospects, reduce reinfection risk, and improve prognosis in complex cases, whereas resin-based sealers, used by general dentists, provide a reliable seal but with lower biological benefits [41].

The most commonly chosen temporary material was glass ionomer. Literature does not clearly indicate whether intra-orifice sealants should be used under final or temporary restorations; nevertheless, no unified or standardized protocol exists [42].

Vital pulp therapy was applied by approximately half of general dental practitioners and by less than half of endodontists, mainly in selected cases such as young patients or accidental pulp exposures. General practitioners are more likely to encounter situations where the pulp is accidentally exposed, for example during deep restorations or managing cracks. In such cases, vital pulp therapy is usually applied to preserve pulp vitality, so it is natural that general practitioners may use this method more frequently [43].

Half of general dental practitioners never perform revitalization in teeth with incomplete root formation, whereas 69 (89.6%) of endodontists do. This biologically based procedure has a clearly defined protocol developed by the European Society of Endodontology (ESE). Although controlled clinical trials are still limited, growing clinical experience indicates that the method is safe and effective. As protocols may change based on new evidence, it is important to follow updates and adhere to the latest guidelines to ensure optimal treatment outcomes [44]. Additionally, 57 (74%) of endodontists never use devitalizing paste, compared to less than half 142 (48.3%) of general practitioners. According to the 2006 ESE guidelines, chemical pulp devitalization with toxic substances is not recommended; the alternative is pulpectomy [4]. The 2023 ESE S3 clinical practice guidelines emphasize the latest evidence on pulp and apical disease diagnosis and treatment efficacy, further highlighting safe and evidence-based clinical strategies [45].

Naturally, most endodontists refer patients to a prosthodontist/therapist for definitive restoration after treatment. When asked about the interval from endodontic treatment to restoration, 172 (46.2%) reported restoration within a few days. An eight-year retrospective study indicates that early restoration (< 2 weeks) is associated with better long-term outcomes [46]. In our country, one-third of general dental practitioners 118 (31.7%) restore teeth after several weeks, which may negatively affect long-term treatment success. Observational studies have shown that effective restoration timing is as soon as possible after endodontic treatment [47].

Both dentists and endodontists most often chose direct composite for restoration, with crowns in second place. However, it is crucial to evaluate the amount of remaining tooth structure. Literature indicates that indirect restorations, especially full-coverage crowns, show better success and longevity due to higher fracture resistance and effective stress distribution. Direct composite restorations are suitable only when sufficient remaining tooth structure exists [48].

Continuous education and professional development of dentists and endodontists are essential factors for ensuring high-quality endodontic treatment. The findings indicate that some specialists still do not use a rubber dam consistently and that the application of vital pulp therapy and current protocols varies, underscoring the need to continuously update knowledge of contemporary endodontic technologies, materials, and clinical protocols. Regular participation in courses, seminars, and review of scientific literature enables practitioners to apply the latest evidence-based strategies, ensure patient safety, and optimize treatment outcomes.

### Limitations

This study has several limitations. Due to the cross-sectional study design, causal relationships cannot be established, and changes in clinical practice over time cannot be assessed. The questionnaire was not formally validated, which may limit the reliability and comparability of the findings. The response rate could not be calculated due to the public dissemination of the survey. The obtained data are based on respondents recruited through online channels and self-reported information, which may not fully reflect actual clinical practice.

## Conclusion

This study showed that endodontic practice in Lithuania has changed in recent years. Dentists are now using more modern, evidence-based methods, such as apex locators, recommended irrigation protocols, and intracanal medicaments. Endodontists tend to follow international guidelines more closely and use advanced treatment techniques more consistently than general dental practitioners.

Despite these improvements, some practices still do not fully align with the recommendations of the European Society of Endodontology (ESE). In particular, the use of rubber dams is not yet routine, even though they are considered a standard of care. The adoption of newer biological approaches, such as vital pulp therapy and regenerative procedures, also varies among practitioners.

In summary, ongoing professional development, adherence to updated guidelines, and evidence-based decision-making remain essential to closing this gap and ensuring high-quality endodontic care.

## Data Availability

The datasets used and/or analysed during the current study are available from the corresponding author on reasonable request.

## Abbreviations

AAE: American Association of Endodontists
ESE: European Society of Endodontology
EDTA: Ethylenediaminetetraacetic acid
NaOCl: Sodium hypochlorite
